# The Interaction between HIV and Intestinal Helminth Parasites Coinfection with Nutrition among Adults in KwaZulu-Natal, South Africa

**DOI:** 10.1155/2017/9059523

**Published:** 2017-03-22

**Authors:** B. T. Mkhize, M. Mabaso, T. Mamba, C. E. Napier, Z. L. Mkhize-Kwitshana

**Affiliations:** ^1^Department of Biomedical and Clinical Technology, Faculty of Health Sciences, Durban University of Technology, Durban, South Africa; ^2^Department of Medical Microbiology, School of Laboratory Medicine and Medical Sciences, College of Health Sciences, University of KwaZulu-Natal, Durban, South Africa; ^3^Epidemiology and Strategic Information Unit, HIV/AIDS, STI and TB (HAST), Human Sciences Research Council, Durban, South Africa; ^4^Department of Food and Nutrition, Faculty of Applied Sciences, Durban University of Technology, Durban, South Africa; ^5^Department of Biomedical Sciences, Faculty of Natural Sciences, Mangosuthu University of Technology, Durban, South Africa

## Abstract

In South Africa few studies have examined the effects of the overlap of HIV and helminth infections on nutritional status. This cross-sectional study investigated the interaction between HIV and intestinal helminths coinfection with nutritional status among KwaZulu-Natal adults. Participants were recruited from a comprehensive primary health care clinic and stratified based on their HIV, stool parasitology, IgE, and IgG4 results into four groups: the uninfected, HIV infected, helminth infected, and HIV-helminth coinfected groups. The nutritional status was assessed using body mass index, 24-hour food recall, micro-, and macronutrient biochemical markers. Univariate and multivariate multinomial probit regression models were used to assess nutritional factors associated with singly and dually infected groups using the uninfected group as a reference category. Biochemically, the HIV-helminth coinfected group was associated with a significantly higher total protein, higher percentage of transferrin saturation, and significantly lower ferritin. There was no significant association between single or dual infections with HIV and helminths with micro- and macronutrient deficiency; however general obesity and low micronutrient intake patterns, which may indicate a general predisposition to micronutrient and protein-energy deficiency, were observed and may need further investigations.

## 1. Background

Approximately 2 billion (24%) of the world's population is infected with intestinal helminth parasites, with high prevalence occurring in poor and deprived communities in tropical and subtropical regions, including sub-Saharan Africa [1]. Helminths may impair the nutritional status in these infected individuals [2]. In sub-Saharan Africa the geographic overlap between the human immunodeficiency virus (HIV), intestinal helminth parasites, and malnutrition may have an additive impact on the competency of the immune system in affected hosts [3, 4]. This triple burden may lead to accelerated HIV and helminth disease progression [5–7]. Potent immune responses and adequate nutrition are essential to resist infectious agents. Research suggests that individuals who are coinfected with HIV and helminths have lower biochemical levels of micronutrients [8], as well as carbohydrate and protein macronutrients [4, 9]. It has been reported that deficiencies of protein, energy, and micronutrients including iron, zinc, and vitamins impact on competent cell mediated and humoral immune responses, and the link to increased susceptibility to HIV and helminth coinfections in such cases has been demonstrated [10, 11]. Thus, micronutrient and macronutrient deficiencies may predispose individuals to HIV and helminth coinfection as well as leading to exacerbated HIV progression, resulting in a vicious cycle of malnutrition, infection, and immune deficiency.

A significant proportion (approximately 54%) of South Africans live under conditions of poverty [12]. Furthermore, KwaZulu-Natal (KZN), a province of South Africa, has a significant proportion of the population living in environments where there is lack of adequate sanitation (22.7%) and safe water supplies (15.8%) [13]. In these areas, the standard of living is generally poor and intestinal helminth infections are highly prevalent [14]. Prevalence of intestinal helminths was found to range among adults from 11.2% in the inland region, 30.3% in the north coast region, and 29.2% in the south coast region [15]. KwaZulu-Natal also has the highest HIV prevalence in South Africa, reported to be 37.4% in 2014 compared to the national estimate of 10.2% [16]. However, despite these data, studies of the possible deleterious effects of HIV and helminth coinfection on nutritional status among adults in KZN are lacking. This study investigated the interaction of HIV and intestinal helminth coinfection with nutritional status as measured by body mass index (BMI) and biochemical micro- and macronutrient markers, against food intake levels, in a periurban informal setting in KZN.

## 2. Methods

### 2.1. Study Setting

The study was conducted in a periurban area, randomly selected from eThekwini enumeration areas under the eThekwini Health District in the KZN province of South Africa. It comprises approximately 39,000 households with approximately 30% informal settlements [17]. Poverty is widespread in this area, with low income households, and approximately 34% of the population in the area were not economically active [17]. There is generally poor access to facilities in the area [18] with about 60% households not having piped water inside the household [17].

The study site was a comprehensive primary health care clinic, providing all essential health care services, including HIV counselling and testing (HCT). Recruitment was therefore purposively conducted in this clinic. By default, the majority of clinic attendees were female.

### 2.2. Recruitment and Selection of Study Participants

Ethical approval to conduct the study was obtained from the University of KwaZulu-Natal Biomedical Research Committee (BREC Ref: BE 230/14). Permission to conduct the study was granted by the Provincial and eThekwini Health District office and the KZN Provincial Department of Health. The local political authorities granted permission to conduct the study in their area, after a series of meetings where the study objectives were explained and discussed.

**Figure 1:**
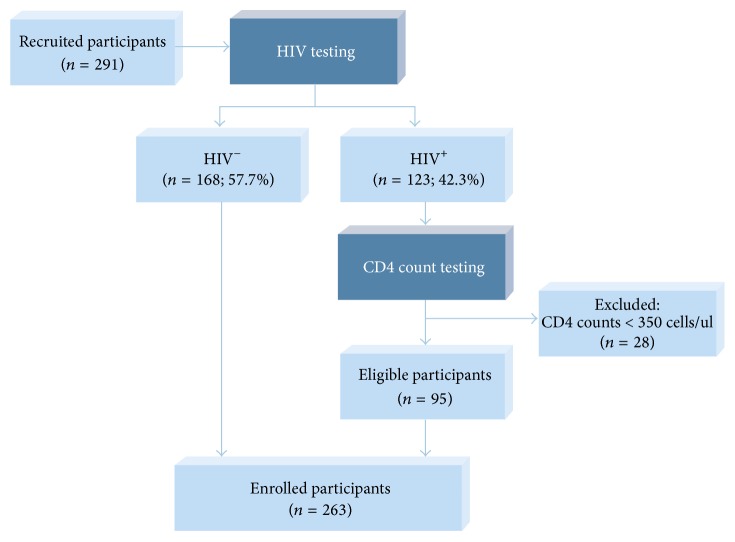
Participant recruitment and enrolment strategy.

During the recruitment process, information sessions were held in the reception area, to inform all the clinic attendees about the study. Those willing to participate were individually given further information. After ensuring that the potential participants fully understood the study, they were asked to give informed consent. They then underwent HIV pretest counselling at the HCT clinic. Eligible participants were adults who were 18 years of age and older, not on antiretroviral therapy, and not pregnant, if female. The enrolment process is outlined in Figure 1.

### 2.3. Ethical Considerations

The study commenced only after ethical approval and permissions from the relevant authorities were obtained. All eligible participants gave written consent before enrolment into the study. Participants were tested for HIV status for the purpose of allocating them to either a study or a reference group. Pre- and post-HIV test counselling was provided. The HIV infected individuals who had CD4 counts below 350 cells/*μ*l were referred to the HCT clinic and were excluded from participating in the study for ethical reasons. The country guidelines recommend the protection of vulnerable individuals such as very sick or severely immunocompromised persons. Likewise, for classifying helminth infection status, participants were screened for intestinal parasites. Those who were found to be infected were referred to the clinic for anthelminthic treatment.

### 2.4. Study Design and Sample Size

A cross-sectional survey of HIV and intestinal helminths prevalence including the investigation of nutritional status was conducted between June 2014 and May 2015 in the eThekwini Health District in KZN. The objective was to describe the nutritional status of individuals infected singly or dually with HIV and intestinal helminths in comparison with noninfected counterparts. A sample size of 229 adults was calculated to detect an effect size of 0.4 with 80% power and probability of 95% between the study groups. The study sample was to include 160 adults not infected with parasites and 69 infected with parasites, assuming that 30% of adults in KZN are infected with parasites, based on the 20.4% prevalence reported on KZN adults [15]. Fifty percent of the study sample would be coinfected with HIV and 50% not be infected with HIV, assuming that 50% of KZN adults are HIV infected, based on the 2011 HIV prevalence of 37.4% among antenatal women in KZN and 2011 HIV prevalence of 38% in the eThekwini district [19].

### 2.5. Measures

Participants were tested for HIV and were screened for intestinal helminth parasites. Demographic data and socioeconomic status data were collected using a structured questionnaire. Nutritional status was assessed using anthropometric measurements, micro- and macronutrient markers, and 24-hour food recall.

#### 2.5.1. Diagnosis of HIV Status

Participants were tested for HIV status using the Alere Determine™ HIV-1/2 Ag/Ab Combo rapid test kit (Orgenics Ltd, Israel). Inconclusive results were confirmed using the Uni-Gold™ Recombigen® HIV-1/2 rapid test kit (Trinity Biotech, Ireland).

#### 2.5.2. Screening for Intestinal Helminth Parasites

Each participant donated stool samples collected on two consecutive days. Upon arrival of the samples in the laboratory, the Kato Katz preparations were made on the same day. A proportion of the sample was then preserved in 10% formol ether in the Mini Parasep tubes (Mini Parasep® Faecal Parasite Concentrator: Apacor Ltd, England) for analysis the following day, by two trained personnel. The stool samples were screened microscopically for intestinal helminth parasites eggs and ova using both the Kato Katz and the modified formol ether (Mini Parasep) methods. However, diagnosis by egg count can be inaccurate [20], when a sample may not contain many eggs, which may be caused by light infections or by day to day variation in egg excretion [21]. Egg excretion depends on immune responses to the parasite infection and genetic and environmental factors [22]. Adams et al. [22] recommended that analyses on the interaction between HIV and helminths should not only be based on the detection or nondetection of eggs in stool samples, since individuals who are infected with parasites in larval stages or male worms only, which cannot produce eggs, may be excluded. Hence, serological diagnosis of intestinal helminths, using* Ascaris*-specific IgE and* Ascaris*-specific IgG4 levels, was done, which supplemented the conventional microscopic diagnosis of helminth infection [20, 22, 23]. Blood samples that were collected from each participant by a trained phlebotomist were assayed for* Ascaris*-specific IgE and* Ascaris*-specific IgG4 levels in a South African National Accreditation System (SANAS) accredited pathology laboratory, using the Phadia® ImmunoCAP method.

*Ascaris*-specific IgE and IgG4 antibodies show cross-reactivity between the antigens of different helminth parasites including* Trichuris trichiura* [24, 25]. Cut-off values of* Ascaris*-specific IgE and* Ascaris*-specific IgG4 were 0.35 kU/l and 0.15 kU/l, respectively, and any levels above the cut-off values were considered high. Infection with intestinal helminths was defined either by the presence of helminth eggs or ova in the stool samples and/or by high levels of* Ascaris*-specific IgE and/or IgG4 in serum.

The participants were stratified, based on the HIV, stool, IgE, and IgG4 results, into four groups: (1) coinfected with HIV and intestinal helminths, (2) infected with only HIV, (3) infected with intestinal helminths only, and (4) not infected.

#### 2.5.3. Nutritional Status

*Anthropometric Measurements*. Weight and height were measured using a calibrated Kern® MPE scale (Kern & Sohn, Germany). The participants were weighed with light clothing, without shoes. The scale calculated and displayed the BMI after the weight and height were keyed into the scale. To determine the BMI (kg/m^2^) of the participants, the cut-off points established by the World Health Organization [26] were used to classify the participants into underweight (<18.5), normal weight (18.5–24.9), overweight (25–29.9), and obese categories (≥30) for both males and females.

*Nutrient Adequacy Ratios (NARS) Analysis for Micro- and Macronutrient Intake*. Trained fieldworkers administered a structured questionnaire to collect 24-hour food recall data from the enrolled participants. Two food recall interviews were conducted to collect data on food items and their quantities, which were consumed the day before the day of the interview by each participant. The first questionnaire was for that which was consumed on a weekday and the second was for that consumed on the weekend. Beverages, regular and special meals, and between-meals snacks consumed, and how they were prepared, were recorded. Three-dimensional food models and a food model booklet were used to indicate food quantities and meal portions. Demographic data indicates that most of the interviewees were the main people in their households who were responsible for the preparing and cooking of meals. Data for the two food recalls were then averaged and nutrient adequacy ratios (NARS) were calculated by a trained nutrition specialist. A nutrient adequacy ratio is the ratio of a nutrient intake divided by the recommended daily requirement for that nutrient [27]. 

*Biochemical Analysis of Micro- and Macronutrients*. Biochemical and haematologic analyses were conducted in a SANAS accredited pathology laboratory. The following biochemical markers of nutrition were analysed by a spectrophotometric autoanalyser: macronutrients: total protein, albumin, and prealbumin; micronutrients: calcium, magnesium, phosphate, zinc, iron, and ferritin. Haemoglobin, haematocrit, white cell count, and differential count levels were assayed with a haematology autoanalyser that uses flow cytometry and sodium lauryl sulphate- (SLS-) haemoglobin methods.

### 2.6. Statistical Analysis

Descriptive statistics was used to summarize the data. Differences between the infected and uninfected groups were assessed using the Kruskal Wallis test for categorical variables and the Wilcoxon signed rank sum test for continuous variables (*p* < 0.001). The outcome variable has four levels: uninfected, HIV singly infected, helminth singly infected, and HIV-helminth coinfected, which is a multinomial outcome. Therefore, univariate and multivariate multinomial probit regression models were used to assess nutritional factors associated with each group (HIV singly infected, helminth singly infected, and HIV-helminth coinfected), and the uninfected group was used as a reference category. Final multivariate models of effects of independent variables associated with each group are presented. Regression coefficients with 95% confidence intervals (CI) are reported to indicate the strength and direction of association and a *p* value ≤ 0.05 to indicate the level of statistical significance. Data was analysed using the statistics packages STATA 12.0 (College Station, Texas, Stata Corporation, USA), SPSS version 23 (IBM Corporation, NY, USA), and GraphPad Prism version 5.01 (GraphPad Software, Inc., USA).

## 3. Results

### 3.1. Characteristics of the Study Participants

#### 3.1.1. Sociodemographic Profile of the Study Participants

Out of a total of 263 enrolled participants (Figure 1), the majority of the participants (91.6%; *n* = 241) were female. The average age of the study participants was 36 years, ranging from 18 to 83 years. The majority were generally poor and 91.3% (*n* = 240) were unemployed. Some relied on government grants, either pension (*n* = 39; 14.9%) or a child support grant (*n* = 93; 35.5%), as their main source of income and 31.2% (*n* = 82) were dependent on their parents for their livelihood. The education level of this population was low, only 3.3% had tertiary education and 67.7% (*n* = 178) had secondary level education, a few up to 12th Grade. About 33% were unable to access clean water; they reported having to share a public tap or use neighbours' taps or tanks and boreholes. Most of the population (54.8%) were using pit latrines while 7.6% reported not having any toilet facilities and some using public mobile toilets. Some participants (25.1%) were using flush toilets which were not connected to sewerage pipes (Table 1).

#### 3.1.2. Nutrient Intake of the Study Population

**Figure 2:**
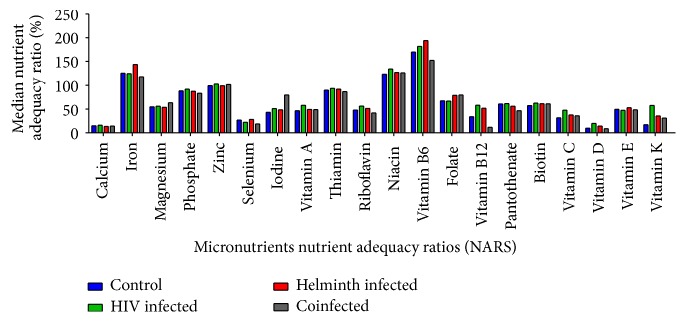
Median nutrient adequacy ratios (NARS) of micronutrients consumed by uninfected, HIV singly infected, helminth singly infected, and HIV-helminth coinfected participant groups.

Nutrient adequacy ratio (NARS) analysis from the 24-hour food recall showed that generally the intake of the micronutrients analysed was similar across all the groups, with the exception of iodine which was highest among the coinfected group and vitamin B12, which was lowest among this group (Figure 2). Further analysis showed that various micronutrient intake levels were lower than the required daily intake (100%) for all the groups, which included calcium, magnesium, selenium, iodine, vitamin A, riboflavin (vitamin B2), pantothenate (vitamin B5), folate, vitamin B12, biotin (vitamin H), vitamin C, vitamin D, vitamin E, and vitamin K. Phosphate, zinc, and thiamin (vitamin B1) were, however, close to the normal required intake. Intake levels for iron, niacin (vitamin B3), and vitamin B6 were higher than the 100% required daily intake for all the participant groups.

**Figure 3:**
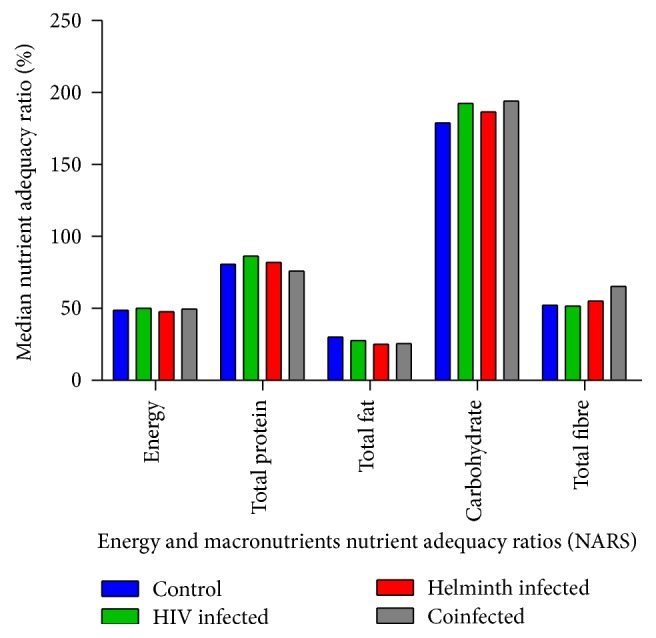
Comparison of median nutrient adequacy ratios (NARS) of macronutrients consumed by the different participant groups.

The macronutrient NARS analysis showed that the coinfected group did not differ from the other groups, where all groups had low levels in all the macronutrient intake levels except for carbohydrates. All the participant groups had a low mean intake (less than the 100% required daily intake) of energy, total protein, total fat, and total fibre (Figure 3). Notably, the intake of carbohydrates was higher than the daily required quantity in all the participant groups, way above 100%, and it was highest in the HIV infected and the coinfected groups (Figure 3).

**Figure 4:**
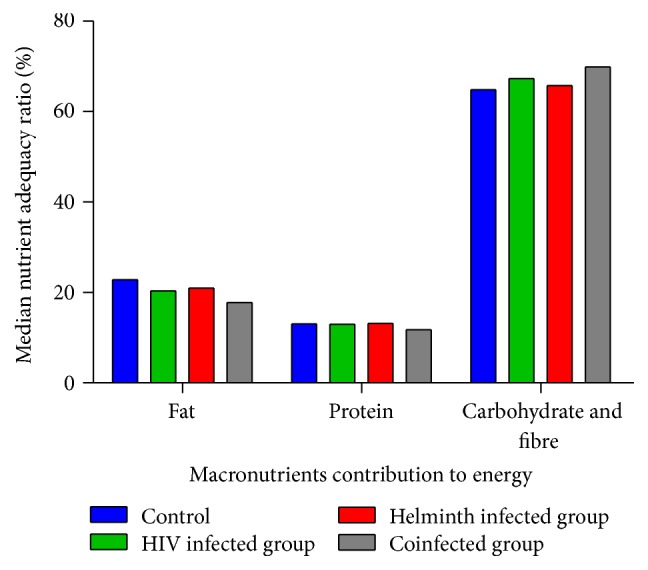
Macronutrient distribution ranges to energy in the different participant groups.

The acceptable macronutrient distribution ranges (AMDR) (fat 15–30%, protein 10–15%, and carbohydrate and fibre 55–75%) to energy showed that all the participant groups had lower contributions of total fat and total protein, less than 30% and 15%, respectively. The contribution of carbohydrate and fibre to energy was within the acceptable range for all participant groups (Figure 4).

### 3.2. Prevalence of HIV and Intestinal Helminths

The overall prevalence of intestinal helminths was 36.1% (*n* = 95) and that of HIV was also 36.1% (*n* = 95). The participants who were singly infected with HIV were 23.6% (*n* = 62), those singly infected with helminths were also 23.6% (*n* = 62), those coinfected with both HIV and intestinal helminths were 12.5% (*n* = 33), and those uninfected with either HIV or helminths were 40.3% (*n* = 106).

The prevalence of helminth infection, determined by microscopic screening for parasite eggs or ova was 11.8% (*n* = 31), where the predominant species was* Ascaris lumbricoides* (*n* = 25; 9.5%), followed by* Trichuris trichiura* (*n* = 6; 2.3%). Furthermore, the serological diagnosis of helminth infection, by high levels of* Ascaris*-specific IgE and IgG4, revealed a prevalence of 29.7% (*n* = 78).

### 3.3. Nutritional Status

#### 3.3.1. Anthropometry

The body mass index (BMI) measures of nutritional status among HIV singly infected, helminth singly infected, HIV-helminth coinfected, and uninfected participants are described in Table 2, showing the differences between the participant groups, although not statistically significant (*p* = 0.089). In the uninfected group 39.0% of the participants were overweight and 51.3% were obese. In the HIV-helminth coinfected group 16.9% were overweight and 5.3% were obese. The helminth singly infected group had 26.3% of participants who were obese. The proportions of participants who were underweight was low (*n* = 6). Of this number, 50% were HIV infected, 33.3% were helminth infected, and only 16.7% were coinfected and none of the uninfected group were underweight.

#### 3.3.2. Biochemical and Haematologic Analysis

The biochemical and haematologic measures of nutritional status among HIV singly infected, helminth singly infected, HIV-helminth coinfected, and uninfected participants are described in Table 2. Except for BMI and phosphate, there was a statistically significant difference in biochemical and haematologic measures across the groups (*p* < 0.001). The median micronutrient levels were varied among the groups although all were within the reference ranges, with transferrin and ferritin levels being lower in the coinfected group compared to the reference group. Percentage transferrin saturation levels were higher in the HIV infected and the coinfected groups compared to the other groups. C-reactive protein, a marker of inflammation, was within range for all the participant groups.

The median biochemical levels of macronutrients (total protein, albumin, and prealbumin) varied among the groups although all were within the reference ranges. Total protein levels were lowest in the uninfected group and highest in both the HIV infected and the HIV-helminth coinfected groups. Albumin levels were lowest in the HIV infected group and highest in the uninfected group. Prealbumin levels were lowest in the HIV infected group and highest in the helminth infected group.

The haematology parameters revealed levels that were within the reference ranges. However, the HIV and the coinfected groups had lower haemoglobin levels compared to the other groups. The absolute eosinophil count levels were highest in the helminth infected group compared to the other groups.

### 3.4. Associations between HIV and Helminth Coinfection and Single Infection with Nutritional Status

The estimated coefficients of the multivariate multinomial probit model are presented in Table 3. BMI was not statistically significant in all the infection groups. Relative to the uninfected group, the HIV-helminth coinfected group was associated with a significant increase in total protein [*β* = 0.16 (0.07–0.25), *p* < 0.001]; and percentage transferrin saturation was also significantly higher [*β* = 0.34 (0.02–0.67), *p* = 0.040] and ferritin significantly decreased [*β* = −0.03 (−0.06–0.01), *p* = 0.006]. The HIV singly infected group was associated with significant increase in total protein [*β* = 0.16 (0.08–0.24), *p* < 0.001] and a significant decrease in albumin [*β* = −0.26 (−0.45–0.08), *p* = 0.005]. The helminth infected group was associated with a significant increase in absolute eosinophils [*β* = 5.07 (1.52–8.63), *p* = 0.005].

## 4. Discussion

In many regions of developing countries, malnutrition is superimposed with endemic helminth and HIV infections. The findings of this study showed that the prevalence of HIV (36.1%) and helminths (36.1%) was high in this adult population (the majority of whom were females), with notable levels of HIV-helminth coinfection. This was against the backdrop of scant data on the prevalence of intestinal parasites in adults of KZN, where most of the prevalence studies have been conducted in schoolchildren. The only other study on prevalence of intestinal helminth parasites in KZN among adults found overall moderate levels of helminth prevalence (20.4%) in the eThekwini district [15]. The higher HIV prevalence in this study is to be expected given the fact that the study site was situated in eThekwini district which has one of the highest HIV prevalence rates in KZN, with a 38% HIV prevalence rate among antenatal women being reported in 2011 for this district [19].

The majority of participants who were obese and overweight (66.3%) were among the uninfected group. Nutrient adequacy ratios analysis revealed a significantly increased carbohydrate intake among all groups, much above the recommended dietary allowance [28]. Increased carbohydrate intake causes weight gain leading to obesity [29]. This may be expected as the general South African population is reported to have a significant proportion of adults who are overweight and obese. The South African National Health and Nutrition examination survey (SANHANES-1) established that 25% and 40.1% of women are overweight and obese, respectively, and 19.6% and 11.6% men are overweight and obese, respectively [30]. This could probably be due to the general consumption of diets that are rich in refined carbohydrates [29, 31] which would be in line with the current study which revealed excessive carbohydrate intake.

Despite the substantially elevated carbohydrate intake in this study population, the energy intake was low. This may be attributed to the fact that fat and protein intake were less than the recommended daily intake. Protein and carbohydrates constitute a lower contribution to energy (16.8 kilojoules per gram each) compared to fat (37 kilojoules per gram) [32, 33], where protein, carbohydrates, fat, and fibre would all together contribute to the required 100%. The contribution of total protein to the daily energy intake was lower than the recommended 15% [34] for all the participant groups. This low protein-energy intake may predispose all the participant groups to protein-energy malnutrition.

The results of anthropometric measurements revealed that underweight was more common among the infected group: 50% among the HIV infected, 33% in the helminth infected group, and 17% in the coinfected group, while none of the uninfected group were underweight. This concurs with the fact that weight loss and wasting is associated with HIV infection and some helminth infections, through a variety of mechanisms including increased energy requirements and/or reduced dietary intake and absorption, reduced appetite, inflammatory cytokines, and diarrhoea [4, 35].

Further analysis showed that the HIV singly infected group was associated with higher total protein and lower albumin biochemical levels compared to the levels of the uninfected group. Similar observations were made in the HIV-helminth coinfected group with regard to significantly higher total protein accompanied by lower albumin levels. Total protein and albumin are serum proteins synthesized by the liver that are not only affected by nutritional status, but by inflammation and infection [36]. Total protein comprises albumin and globulin fractions. Albumin in healthy individuals is highest in concentration in serum, usually 60% of the total protein [37]. HIV infection induces a nonspecific expansion of the globulin fraction due to the polyclonal stimulation of B cells in response to the acute or chronic stages of the infection and associated opportunistic infections [38, 39]. Thus, the higher total protein seen in both the HIV singly infected group and the HIV-helminth infected group may have been as a result of prioritization in the formation of globulins and acute phase proteins in response to the HIV infection [37] and, proportionately, reduced albumin levels. On the other hand, lower albumin levels may have been due to the increased rate of transcapillary leak of albumin into the interstitial fluid associated with infection [40]. Both HIV and helminth infections have acute and chronic stages, resulting in chronic activation of the immune system. However, in this study, it was not possible to determine the stages of both HIV and helminths as this was not within the scope of the study objective. The low albumin finding in the current study is corroborated by a similar finding in the Kannangai et al. [41] study of HIV infected individuals, where albumin levels were low as well.

The food recall NARS analysis also revealed a general low micronutrient intake where the median intake of calcium, magnesium, selenium, iodine, vitamin A, vitamin B2, vitamin B5, vitamin B12, vitamin C, vitamin D, vitamin E, vitamin H, vitamin K, and folate micronutrients was low for all the participant groups. The expected finding would be similarly low biochemical levels since intake levels were low [42]. It had been hypothesized that all the micronutrient biochemical levels would be low in the singly and coinfected infected with HIV and helminths study groups. HIV infection has been reported to predispose to micronutrient deficiency [43] and, likewise, helminth infections have been associated with deficiency of most of the micronutrients [44]. However, in this study, discrepant results were found where biochemical analyses showed that these micronutrients were within the reference range for all the participant groups. Biochemical markers as an indication of nutritional status are more reliable than food intake questionnaire data, and food intake data should be used as evidence of food variety rather than to indicate nutritional status [45, 46]. This discrepancy could have been due to underreported 24-hour food recall data as self-reported actual food intake may have been omitted consciously or by accident, leading to the discrepancy between infection status and biochemical micronutrient levels [47, 48] or the participants may have been taking supplements and failed to declare these during the collection of data [49]. Food recall data collected over 3 days, accompanied by a food frequency questionnaire, may have given a more holistic indication of dietary consumption [50, 51].

Further analysis showed that the HIV-helminth coinfected group was associated with significantly lower ferritin levels, although percentage of transferrin saturation levels were higher with nonstatistically significant lower transferrin levels. Low ferritin levels are typical of iron deficiency anaemia [52]. However, iron intake levels were higher than the daily required quantity for all the participant groups. Intestinal parasitic helminths are associated with iron deficiency anaemia [9, 53] and HIV on its own is also associated with iron deficiency anaemia [54]. Intestinal helminths source nutrients from the host for their own growth, while the infection itself, either caused by HIV or helminths, may increase the host's need for nutrients [55]. Thus, the lower ferritin levels in the coinfected group may indicate subclinical iron deficiency. Subclinical iron deficiency, even though it may be mild, impacts on the physiological functions that drive the development of cells and their metabolic function, which would have an effect on the immune system action against the HIV-helminth coinfection [56]. Moreover, anaemia in the HIV-helminth coinfection may lead to increased HIV progression, increased mortality, and poor quality of life [57]. Mupfasoni et al. [58], in Rwanda, however, found no association of intestinal helminth parasite infection with anaemia, although the authors attributed this to the fact that anaemia was uncommon in their study area.

Although the eosinophil counts were within range for all the groups, the helminth infected and the dually infected groups had significantly higher levels compared to the other groups. These results are in keeping with the classic feature of helminthiasis. These infections are associated with increased production of eosinophils [59, 60], which are reported to decrease significantly after deworming [61, 62].

There was no significant association observed between HIV-helminth coinfection and single infections with micro- and macronutrient deficiency. However, the results highlighted the various micro- and macronutrient intake patterns in the population. Low intake levels of calcium, magnesium, selenium, iodine, vitamin A, vitamin B2, vitamin B5, folate, vitamin B12, biotin (vitamin H), vitamin C, vitamin D, vitamin E, vitamin K, total protein, and energy were noted in all the participant groups. This may indicate a general predisposition to micronutrient and protein-energy deficiency in the study participants and may need further nutritional investigations.

## 5. Limitations of This Study

The cross-sectional design in this study is limited to determining the associations only and cannot infer causality. A prospective cohort study design with randomised sampling would be recommended for such an investigation. The small sample size may have resulted in the inability to determine a significant association between macro- and micronutrient levels and the coinfection. Moreover, the use of self-reported food recall data collected over two days, which relies on memory and correct estimations of quantity, is a limitation, although the value of the data is recognised since it indicated the food intake patterns in the population. Energy intake of the study participants was only about 50% of the reference nutrient intake. However, the prevalence of overweight and obesity was almost two-thirds. Therefore the energy intake may be underestimated. The fat intake may be underestimated as the 24-hour dietary recall may not cover the cooking oil intake. This could result in inaccurate macronutrient contribution to energy. In addition, the study used biased sampling since recruitment was from individuals who attended the HCT clinic and thus the findings cannot be generalised to the population in the area where the study was conducted. Furthermore, the fact that the stool samples were screened microscopically for intestinal helminth parasites eggs and ova the following day is a limitation, although they were prepared and preserved on the same day of collection. This could have significantly affected the ability to detect hookworm eggs since these rapidly disintegrate upon storage of stools. Nevertheless, this study adds value to the less studied but growing research area of HIV-helminth infection impact on nutritional status in sub-Saharan Africa.

## 6. Concluding Remarks

Helminth infection is a neglected disease globally, with more attention and priority given to HIV/AIDS, TB, and malaria. The high prevalence of helminth infection observed in this adult population warrants attention, especially since HIV is endemic in the area. However, there was no significant association between single and dual HIV and helminth infections with micro- and macronutrient deficiency in this population. The frequent occurrence of obesity and overweight which is an additional health burden in South Africa, possibly due to excessive carbohydrate intake, and the general low intake of micro- and protein-energy macronutrients observed in this study require further nutritional investigations and the current South African Department of Health campaign on healthy lifestyle needs strengthening [63]. Future studies should investigate the nutritional, parasitic, and infectious conditions that may act as cofactors for rapid progression of HIV infection [64].

## Figures and Tables

**Table 1:** Demographic and socioeconomic data of the study participants.

Variable	*n* (%)
*N*	263
Gender	
Male	22 (8.4)
Female	241 (91.6)
Age (years)	
18–24	88 (33.5)
25–49	116 (44.1)
≥50	58 (22.1)
Unknown	1 (0.4)
Marital status	
Single	219 (83.6)
Married	32 (12.2)
Widowed	11 (4.2)
Education level	
None	24 (9.2)
Primary	52 (19.8)
Secondary	178 (67.7)
Tertiary	8 (3.3)
Employment	
Unemployed	240 (91.3)
Employed	23 (8.7)
Source of income	
No income	8 (3.1)
Child support grant	93 (35.5)
Pension	39 (14.9)
Dependent	82 (31.2)
Self-employed	19 (7.3)
Salary	16 (6.1)
Salary and second income	5 (1.9)
Source of water	
Tap inside the house	144 (54.8)
Tap outside the house	32 (12.2)
Public tap	66 (25.1)
Tank	16 (6.1)
Neighbour's tap	4 (1.5)
Borehole	1 (0.4)
Toilet facility	
Pit	144 (54.8)
Flush, connected to sewage pipes	33 (12.5)
Flush, not connected^*∗*^	66 (25.1)
Mobile	17 (6.5)
None	3 (1.1)

^*∗*^Flush, not connected: toilets can flush; however, they dispose sewage into septic tanks and the waste is collected once in a while.

**Table 2:** Anthropometric, biochemical, and haematologic measures of nutritional status among HIV singly infected, helminth singly infected, and HIV-helminth coinfected participants.

Variables	Reference ranges	Uninfected (*n* = 106)	HIV infected (*n* = 62)	Helminth infected (*n* = 62)	HIV-helminth coinfected (*n* = 33)	
Body mass index	kg/m^2^	*n* (%)	*n* (%)	*n* (%)	*n* (%)	*p* value

Under-weight	<18.5	0 (0.0)	3 (50. 0)	2 (33.3)	1 (16.7)	0.089^*∗*^
Normal weight	18.5–24.9	35 (34.7)	28 (27.7)	24 (23.8)	14 (13.9)	
Overweight	25.0–29.9	30 (39.0)	18 (23.4)	16 (20.8)	13 (16.9)	
Obese	≥30.0	39 (51.3)	13 (17.1)	20 (26.3)	4 (5.3)	

Nutritional status	Reference ranges	Median (IQR)	Median (IQR)	Median (IQR)	Median (IQR)	*p* value

Total protein (g/l)	60–85	73.0 (71.0–78.0)	78.0 (74.0–85.0)	75.0 (72.0–78.0)	78.0 (75.0–82.0)	<0.001
Albumin (g/l)	35–52	40.0 (37.0–42.0)	37.0 (36.0–40.0)	39.0 (37.0–42.0)	39.0 (37.0–41.0)	<0.001
Prealbumin (mg/dl)	18–38	21.8 (18.4–25.3)	20.9 (17.9–25.4)	23.5 (19.3–26.8)	21.1 (17.8–22.6)	<0.001
Iron (umol/l)	6.6–28.0	12.0 (8.0–16.0)	13.0 (9.0–17.0)	13.0 (9.0–18.0)	12.0 (8.0–17.0)	<0.001
Transferrin (g/l)	1.85–4.05	2.77 (2.45–3.11)	2.63 (2.38–2.96)	2.74 (2.48–3.07)	2.62 (2.20–2.94)	<0.001
% Transferrin saturation	20–55	20.0 (13.0–26.0)	23.5 (18.0–29.0)	21.0 (14.0–29.0)	22.0 (13.0–27.0)	<0.001
Ferritin (ng/ml)	30–400	48.9 (24.1–76.9)	38.5 (23.4–75.0)	44.4 (25.2–73.4)	40.5 (18.4–49.3)	<0.001
Calcium (mmol/l)	2.15–2.62	2.31 (2.25–2.37)	2.30 (2.22–2.36)	2.34 (2.28–2.40)	2.32 (2.23–2.38)	<0.001
Magnesium (mmol/l)	0.65–1.05	0.89 (0.84–0.95)	0.88 (0.83–0.92)	0.88 (0.81–0.94)	0.89 (0.81–0.96)	<0.001
Phosphate (mmol/l)	0.80–1.55	1.08 (0.94–1.22)	1.09 (0.97–1.20)	1.12 (0.97–1.28)	1.14 (0.99–1.27)	0.098^*∗*^
Zinc (umol/l)	9.2–18.4	11.55 (9.1–13.7)	12.7 (10.5–16.3)	11.95 (9.80–14.50)	12.3 (9.6–14.8)	<0.001
C-reactive protein (mg/l)	0–5	3.85 (1.05–9.25)	2.60 (0.00–7.20)	3.10 (0.00–6.10)	3.00 (1.00–6.50)	<0.001
Haemoglobin (g/dl)	12.3–15.3	12.6 (11.9–13.5)	12.3 (11.7–13.3)	12.8 (12.0–13.5)	12.4 (11.8–13.4)	<0.001
Haematocrit (l/l)	0.4–0.56	0.39 (0.36–0.41)	0.38 (0.36–0.40)	0.39 (0.37–0.41)	0.39 (0.35–0.40)	<0.001
White cell count (×10^9^/l)	4.0–10.0	5.99 (4.96–7.07)	5.19 (4.35–6.15)	6.46 (5.58–8.37)	6.69 (4.95–8.48)	<0.001
Absolute neutrophils (×10^9^/l)	2.0–7.5	2.97 (2.29–3.89)	2.29 (1.71–3.03)	3.37 (2.44–4.69)	3.25 (2.37–4.50)	<0.001
Absolute lymphocytes (×10^9^/l)	1.5–4.0	2.22 (1.87–2.58)	2.00 (1.71–2.40)	2.34 (1.85–2.79)	2.35 (1.85–2.74)	<0.001
Absolute eosinophils (×10^9^/l)	0.04–0.4	0.12 (0.08–0.22)	0.12 (0.08–0.25)	0.24 (0.12–0.42)	0.23 (0.09–0.49)	<0.001
Absolute basophils (×10^9^/l)	0.00–0.10	0.02 (0.01–0.02)	0.01 (0.01–0.02)	0.02 (0.02–0.03)	0.02 (0.01–0.03)	<0.001

^*∗*^ *p* > 0.01; IQR, interquartile range specifying first quantile (q25%) and third quantile (q75%).

**Table 3:** Multivariate multinomial probit model of nutritional factors associated with HIV singly infected, helminth singly infected, and HIV-helminth coinfected groups (base category: uninfected group).

Variables	HIV infected (*n* = 62)			Helminth infected (*n* = 62)			HIV-helminth coinfected (*n* = 33)		
	*β*	95% CI	*p* value	*β*	95% CI	*p* value	*β*	95% CI	*p* value
*Body mass index*									
Underweight	Ref			Ref			Ref		
Normal weight	15.90	1629.81–1598.01	0.985	15.02	1628.92–1598.89	0.985	16.59	1630.50–1597.31	0.984
Overweight	16.32	1630.23–1597.589	0.984	15.23	1629.17–1598.65	0.985	16.23	1630.14–1597.68	0.984
Obese	16.98	1630.89–1596.926	0.984	15.70	1629.61–1598.21	0.985	17.63	1631.54–1596.28	0.983
*Nutritional status*									
Total protein (g/l)	0.16	(0.08–0.24)	<0.001	−0.01	(−0.10–0.07)	0.747	0.16	(0.07–0.25)	<0.001
Albumin (g/l)	−0.26	(−0.45–0.08)	0.005	−0.16	(−0.33–0.01)	0.065	−0.12	(−0.35–0.10)	0.288
Prealbumin (mg/dl)	0.10	(−0.01–0.21)	0.078	0.08	(−0.02–0.18)	0.105	−0.03	(−0.17–0.11)	0.656
Iron (umol/l)	0.00	(−0.34–0.34)	0.992	0.06	(−0.19–0.31)	0.628	−0.49	(−1.03–0.05)	0.073
Transferrin (g/l)	−0.26	(−1.78–1.25)	0.735	−0.08	(−1.33–1.17)	0.895	0.18	(−1.91–2.27)	0.865
% Transferrin saturation	0.07	(−0.15–0.28)	0.531	−0.01	(−0.18–0.15)	0.879	0.34	(0.02–0.67)	0.040
Ferritin (ng/ml)	−0.01	(−0.02–0.00)	0.102	0.00	(−0.01–0.00)	0.399	−0.03	(−0.06–0.01)	0.006
Calcium (mmol/l)	0.13	(−4.97–5.22)	0.961	5.32	(−2.50–11.13)	0.073	0.75	(−6.25–7.75)	0.833
Magnesium (mmol/l)	−3.36	(−8.62–1.90)	0.210	−2.60	(−7.40–2.19)	0.287	0.11	(−6.05–6.27)	0.971
Phosphate (mmol/l)	1.28	(−0.99–3.55)	0.268	−0.14	(−2.15–1.87)	0.891	0.39	(−2.32–3.10)	0.779
Zinc (umol/l)	0.07	(−0.03–0.17)	0.149	0.05	(−0.04–0.14)	0.237	0.00	(−0.12–0.11)	0.934
Haemoglobin (g/dl)	−0.12	(−1.29–1.05)	0.841	−0.49	(−1.61–0.63)	0.390	0.23	(−1.28–1.74)	0.765
Haematocrit (l/l)	2.89	(−38.99–44.77)	0.892	21.55	(−18.19–61.29)	0.288	−0.38	(−55.10–54.33)	0.989
White cell count (×10^9^/l)	0.92	(−2.07–3.92)	0.546	−2.27	(−5.22–0.68)	0.132	1.07	(−2.66–4.80)	0.572
Absolute neutrophils (×10^9^/l)	−1.18	(−4.32–1.95)	0.459	2.63	(−0.48–5.73)	0.097	−0.74	(−4.65–3.18)	0.712
Absolute lymphocytes (×10^9^/l)	−1.49	(−4.68–1.70)	0.359	2.42	(−0.77–5.61)	0.137	−0.99	(−4.96–2.97)	0.623
Absolute eosinophils (×10^9^/l)	−1.53	(−5.68–2.62)	0.469	5.07	(1.52–8.63)	0.005	2.43	(−1.90–6.76)	0.272
Absolute basophils (×10^9^/l)	−22.77	(−62.67–17.13)	0.263	0.53	(−32.59–33.65)	0.975	−24.12	(−66.90–18.67)	0.269

## References

[1] World Health Organization Soil-transmitted helminth infections: fact sheet. http://www.who.int/mediacentre/factsheets/fs366/en/.

[2] Gedle D., Gelaw B., Muluye D., Mesele M. (2015). Prevalence of malnutrition and its associated factors among adult people living with HIV/AIDS receiving anti-retroviral therapy at Butajira Hospital, southern Ethiopia. *BMC Nutrition*.

[3] Assefa S., Erko B., Medhin G., Assefa Z., Shimelis T. (2009). Intestinal parasitic infections in relation to HIV/AIDS status, diarrhea and CD4 T-cell count. *BMC Infectious Diseases*.

[4] Koethe J. R., Heimburger D. C. (2010). Nutritional aspects of HIV-associated wasting in sub-Saharan Africa. *American Journal of Clinical Nutrition*.

[5] Amare B., Moges B., Mulu A., Yifru S., Kassu A. (2015). Quadruple burden of HIV/AIDS, tuberculosis, Chronic intestinal parasitoses, and multiple micronutrient deficiency in ethiopia: a summary of available findings. *BioMed Research International*.

[6] Borkow G., Bentwich Z. (2004). Chronic immune activation associated with chronic helminthic and human immunodeficiency virus infections: role of hyporesponsiveness and anergy. *Clinical Microbiology Reviews*.

[7] Karp C. L., Auwaerter P. G. (2007). Coinfection with HIV and tropical infectious diseases: II. Helminthic, fungal, bacterial, and viral pathogens. *Clinical Infectious Diseases*.

[8] Moreau E., Chauvin A. (2010). Immunity against helminths: interactions with the host and the intercurrent infections. *Journal of Biomedicine and Biotechnology*.

[9] Katona P., Katona-Apte J. (2008). The interaction between nutrition and infection. *Clinical Infectious Diseases*.

[10] Chandra R. K. (1997). Nutrition and the immune system: an introduction. *American Journal of Clinical Nutrition*.

[11] Schaible U. E., Kaufmann S. H. E. (2007). Malnutrition and infection: complex mechanisms and global impacts. *PLoS Medicine*.

[12] Statistics South Africa (2014). *Poverty Trends in South Africa: An Examination of Absolute Poverty between 2006 and 2011*.

[13] Statistics South Africa (2015). *Statistical Release P0318: General Household Survey*.

[14] Kamau P., Aloo-Obudho P., Kabiru E. (2012). Prevalence of intestinal parasitic infections in certified food-handlers working in food establishments in the City of Nairobi, Kenya. *Journal of Biomedical Research*.

[15] Kwitshana Z. L., Tsoka J. M., Mabaso M. L. H. (2008). Intestinal parasitic infections in adult patients in KwaZulu-Natal. *South African Medical Journal*.

[16] Statistics South Africa (2015). *Statistical Release P0302: Mid-Year Population Estimates 2015*.

[17] Statistics South Africa http://www.statssa.gov.za/?page_id=4286&id=10333.

[18] Mottiar S., Naidoo O., Khumalo D. (2011). Women's organisations and the struggle for water and sanitation services in Chatsworth and Inanda, Durban: the Westcliff Flats Residents Association and the Didiyela Women's Group. *Agenda*.

[19] Department of Health South Africa (2012). *The National Antenatal Sentinel HIV and Syphilis Prevalence Survey in South Africa*.

[20] Mkhize-Kwitshana Z. L., Taylor M., Jooste P., Mabaso M. L. H., Walzl G. (2011). The influence of different helminth infection phenotypes on immune responses against HIV in co-infected adults in South Africa. *BMC Infectious Diseases*.

[21] Hall A., Hewitt G., Tuffrey V., De Silva N. (2008). A review and meta-analysis of the impact of intestinal worms on child growth and nutrition. *Maternal and Child Nutrition*.

[22] Adams V. J., Markus M. B., Kwitshana Z. L. (2006). Recall of intestinal helminthiasis by HIV-infected South Africans and avoidance of possible misinterpretation of egg excretion in worm/HIV co-infection analyses. *BMC Infectious Diseases*.

[23] Maizels R. M., Yazdanbakhsh M. (2003). Immune regulation by helminth parasites: cellular and molecular mechanisms. *Nature Reviews Immunology*.

[24] Pritchard D. I., Quinnell R. J., McKean P. G. (1991). Antigenic cross-reactivity between Necator americanus and Ascaris lumbricoides in a community in Papua New Guinea infected predominantly with hookworm. *Transactions of the Royal Society of Tropical Medicine and Hygiene*.

[25] Figueiredo C. A., Barreto M. L., Rodrigues L. C. (2010). Chronic intestinal helminth infections are associated with immune hyporesponsiveness and induction of a regulatory network. *Infection and Immunity*.

[26] World Health Organization (1995). *Physical status: the use and interpretation of anthropometry: report of a WHO expert committee*.

[27] Steyn N. P., Nel J. H., Nantel G., Kennedy G., Labadarios D. (2006). Food variety and dietary diversity scores in children: are they good indicators of dietary adequacy?. *Public Health Nutrition*.

[28] National Academy of Sciences (1998). *Dietary Reference Intakes (DRIs): Estimated Average Requirements*.

[29] Van Dam R. M., Seidell J. C. (2007). Carbohydrate intake and obesity. *European Journal of Clinical Nutrition*.

[30] Shisana O., Labadarios D., Rehle T. (2013). *SANHANES-1 Team. South African National Health and Nutrition Examination Survey (SANHANES-1)*.

[31] Pacanaro C. P., Dias S. R., Serafim L. R. (2014). Evaluation of biochemical, hematological and parasitological parameters of protein-deficient hamsters infected with *Ancylostoma ceylanicum*. *PLOS Neglected Tropical Diseases*.

[32] World Health Organization (2005). *Dietary Intake of Fruit and Vegetables and Management of Body Weight*.

[33] Nutrition Information Centre University of Stellenbosch (NICUS) (2010). *NICUS Factsheet: Fats and Oils: Choose Sensibly*.

[34] World Health Organization (2003). *WHO Technical Report Series 916: Diet, Nutrition and the Prevention of Chronic Diseases*.

[35] Broadhurst C., Wilson K. (2001). Immunology of delirium: new opportunities for treatment and research. *British Journal of Psychiatry*.

[36] Harris D., Haboubi N. (2005). Malnutrition screening in the elderly population. *Journal of the Royal Society of Medicine*.

[37] Bishop M. L., Fody E. P., Schoeff L. E. (2005). *Clinical Chemistry: Principles, Procedures, Correlations*.

[38] Fiorino A. S., Atac B. (1997). Paraproteinemia, plasmacytoma, myeloma and HIV infection. *Leukemia*.

[39] Adedeji A. L., Adenikinju R. O., Ajele J. O., Olawoye T. L. (2014). Serum protein electrophoresis under effective control of HIV-1 disease progression. *Experimental and Clinical Sciences*.

[40] Shenkin A. (2006). Serum prealbumin: is it a marker of nutritional status or of risk of malnutrition?. *Clinical Chemistry*.

[41] Kannangai R., Kandathil A. J., Ebenezer D. L. (2008). Usefulness of alternate prognostic serum and plasma markers for antiretroviral therapy for human immunodeficiency virus type 1 infection. *Clinical and Vaccine Immunology*.

[42] Damms-Machado A., Weser G., Bischoff S. C. (2012). Micronutrient deficiency in obese subjects undergoing low calorie diet. *Nutrition Journal*.

[43] Semba R. D., Tang A. M. (1999). Micronutrients and the pathogenesis of human immunodeficiency virus infection. *British Journal of Nutrition*.

[44] Arinola G. O., Morenikeji O. A., Akinwande K. S. (2015). Serum micronutrients in helminth-infected pregnant women and children: suggestions for differential supplementation during anti-helminthic treatment. *Annals of Global Health*.

[45] Shim J.-S., Oh K., Kim H. C. (2014). Dietary assessment methods in epidemiological studies. *Epidemiology and Health*.

[46] Moghames P., Hammami N., Hwalla N. (2016). Validity and reliability of a food frequency questionnaire to estimate dietary intake among Lebanese children. *Nutrition Journal*.

[47] Lasheras C., González C., García A., Patterson A. M., Fernández S. (1999). Dietary intake and biochemical indicators of nutritional status in an elderly institutionalized and non-institutionalized population. *Nutrition Research*.

[48] Streppel M. T., de Vries J. H. M., Meijboom S. (2013). Relative validity of the food frequency questionnaire used to assess dietary intake in the Leiden Longevity Study. *Nutrition Journal*.

[49] Jacques P. F., Sulsky S. I., Sadowski J. A., Phillips J. C. C., Rush D., Willett W. C. (1993). Comparison of micronutrient intake measured by a dietary questionnaire and biochemical indicators of micronutrient status. *American Journal of Clinical Nutrition*.

[50] Yang Y. J., Kim M. K., Hwang S. H., Ahn Y., Shim J. E., Kim D. H. (2010). Relative validities of 3-day food records and the food frequency questionnaire. *Nutrition Research and Practice*.

[51] De Keyzer W., Huybrechts I., De Vriendt V. (2011). Repeated 24-hour recalls versus dietary records for estimating nutrient intakes in a national food consumption survey. *Food and Nutrition Research*.

[52] Cook J. D. (2005). Diagnosis and management of iron-deficiency anaemia. *Best Practice and Research: Clinical Haematology*.

[53] Crompton D. W. T., Nesheim M. C. (2002). Nutritional impact of intestinal helminthiasis during the human life cycle. *Annual Review of Nutrition*.

[54] Volberding P. A., Levine A. M., Dieterich D., Mildvan D., Mitsuyasu R., Saag M. (2004). Anemia in HIV infection: clinical impact and evidence-based management strategies. *Clinical Infectious Diseases*.

[55] Stephensen C. B. (1999). Burden of infection on growth failure. *Journal of Nutrition*.

[56] Bhaskaram P. (2001). Immunobiology of mild micronutrient deficiencies. *British Journal of Nutrition*.

[57] Akinbo F. O., Okaka C. E., Omoregie R. (2011). Prevalence of intestinal parasites in relation to CD4 counts and anaemia among HIV-infected patients in Benin City, Edo State, Nigeria. *Tanzania Journal of Health Research*.

[58] Mupfasoni D., Karibushi B., Koukounari A. (2009). Polyparasite helminth infections and their association to anaemia and undernutrition in Northern Rwanda. *PLoS Neglected Tropical Diseases*.

[59] Maizels R. M., Balic A. (2004). Resistance to helminth infection: the case for interleukin-5-dependent mechanisms. *Journal of Infectious Diseases*.

[60] Bethony J., Brooker S., Albonico M. (2006). Soil-transmitted helminth infections: ascariasis, trichuriasis, and hookworm. *Lancet*.

[61] Hagel I., Lynch N. R., Di Prisco M. C., Rojas E., Perez M., Alvarez N. (1993). Ascaris reinfection of slum children: relation with the IgE response. *Clinical and Experimental Immunology*.

[62] Mulu A., Anagaw B., Gelaw A., Ota F., Kassu A., Yifru S. (2015). Effect of deworming on Th2 immune response during HIV-helminths co-infection. *Journal of Translational Medicine*.

[63] Department of Health South Africa Strategic Plan 2014/2015–2018/2019. https://www.health-e.org.za/wp-content/uploads/2014/08/SA-DoH-Strategic-Plan-2014-to-2019.pdf.

[64] Stillwaggon E. (2009). Complexity, cofactors, and the failure of AIDS policy in Africa. *Journal of the International AIDS Society*.

